# 313 Data-Driven Evaluation of Community Health Worker Program – CORRIGENDUM

**DOI:** 10.1017/cts.2024.522

**Published:** 2024-04-23

**Authors:** Roselyne Tchoua, Kate Karam, Kelly McCabe

The above abstract published with an incorrect title.

The incorrect title was “313 Identification of novel plasma protein of Community Health Worker Program”, the correct title is “313 Data-Driven Evaluation of Community Health Worker Program”.

The original abstract has been corrected online to rectify this error.
